# 'Personal Care' and General Practice Medicine in the UK: A qualitative interview study with patients and General Practitioners

**DOI:** 10.1186/1750-4732-1-13

**Published:** 2007-08-31

**Authors:** Rachel Adam

**Affiliations:** 1 Centre for Research on Families and Relationships, the University of Edinburgh, 23 Buccleuch Place, Edinburgh, Scotland, UK

## Abstract

**Background:**

Recent policy and organisational changes within UK primary care have emphasised graduated access to care, speed of access to the first available general practitioner (GP) and care being provided by a range of healthcare professionals. These trends have been strengthened by the current GP contract and Quality and Outcomes Framework (QOF). Concern has been expressed that the potential for personal care is being diminished as a result and that this will reduce quality standards. This paper presents data from a study that explored with patients and GPs what personal care means and whether it has continuing importance to them.

**Methods:**

A semi-structured questionnaire was used to interview participants and Framework Analysis supported analysis of emerging themes. Twenty-nine patients, mainly women with young children, and twenty-three GPs were interviewed from seven practices in Lothian, Scotland, ranged by practice size and relative deprivation score.

**Results and Discussion:**

Personal care was defined mainly, though not exclusively, as care given within the context of a continuing relationship in which there is an interpersonal connection and the doctor adopts a particular consultation style. Defined in this way, it was reported to have benefits for both health outcomes and patients' experience of care. In particular, such care was thought to be beneficial in attending to the emotions that can be elicited when seeking and receiving health care and in enabling patients to be known by doctors as legitimate seekers of care from the health service. Its importance was described as being dependent upon the nature of the health problem and patients' wider familial and social circumstances. In particular, it was found to provide support to patients in their parenting and other familial caring roles.

**Conclusion:**

Personal care has continuing salience to patients and GPs in modern primary care in the UK. Patients equate the experience of care, not just outcomes, with high quality care. As it is mainly conceptualised and experienced as care within the context of a continuing relationship, policies and organisational arrangements that support and give incentives to this must be in place. These preferences are not strongly reflected in the QOF. Specific questions need to be addressed by future audit and research on the impact of the contract on these aspects of service.

## Background

Providing 'personal care', focused around the needs of patients, families and communities, is of central importance to general practice and primary care services more generally in many Western countries. In the UK, there is a long tradition of the personal aspect of care being at the heart of General Practitioners' (GPs') professional vocation. It is a concept that is deeply rooted in the tradition of General Practice and pervades its professional rhetoric and literature. The discourse of the personal doctor caring for the whole family from cradle to grave or, at the very least, through an episode of illness is recurrently invoked. For many, the archetypal Dr. Findlay providing personal care to patients is the quintessential General Practitioner [1,2]. Providing such care also has been linked to statements of what constitutes high quality standards [3,4] and to how professional practice should be shaped in the modern world [5].

It is well recognized that what constitutes personal care has been conceptualized in a number of different ways. These conceptualisations have tended to be academic- and researcher-led rather than being defined by patients or frontline service providers. The idea of the personal in care is implicit in work on, for instance, the 'therapeutic' patient-doctor relationship, 'continuity of care' with the same healthcare professional over time or continuity of patient information, the 'patient-centred' consultation style, the 'patient-centred clinical method', and in other ideas like patient enablement and empowerment in which the idea of 'patient as person' has primacy [3,6-8].

Regardless of its stated importance, organisational changes of UK primary care that have emphasised graduated access to care, including telephone services, walk-in clinics and greater use of community pharmacists may have been implemented at the expense of more personal components of care that emphasise a patient-doctor relationship based on mutual trust and personal attention [9,10]. The most recent, and some would say radical, of these changes are underpinned by the contract for General Practice implemented in 2004. A national system of financial rewards in the form of the Quality and Outcomes Framework (QOF) was introduced. Changes brought in or strengthened by the contract that may influence the delivery of personal care include: patients being registered with a practice rather than individual GPs; emphasis on speed of access to *any *rather than with one healthcare practitioner; larger practices offering multi-professional care; GP specialization; and changes in the organisation of care that reflect the emphasis on the prevention, detection and management of chronic diseases.

As described by Martin [11], the QOF is based on a complex points system which translates into financial remuneration. The framework covers three sections; clinical indicators, organisational indicators and patient experience, each of which has criteria for quality standards that attract quality-of-care payments. There is concern that, as the framework currently stands, the incentives inherent in this framework will further undermine the potential for personal care in primary care services and may disadvantage the neediest patients [12]. Some have argued that the contract gives priority to a more public health, or what might be termed a biomedical, 'population' level approach to medicine, which threatens GPs' ability to offer more personal, patient-centred and individualized care. It is the 'spirit', as well as the reality, of the personal doctor that is thought to be at risk of dying.

Despite the strength and currency of this debate, what is lacking is empirical evidence of whether the concept of personal care continues to have salience to patients and doctors in the twenty-first century. How is it conceptualised, particularly by those using services and those at the frontline of provision? Is it valued by and beneficial to them? Does this vary depending on patients' health status or other circumstances, for instance, when parenting children or in other familial caring roles?

One of the few studies in the UK which explored how patients and healthcare professionals defined and valued personal care found that such care is promoted by, but does not always rely upon, that provided in the context of a continuing relationship [13,14], what is now commonly being termed *relational continuity*. It suggests that what makes care personal is not dependent on a relationship with one provider as such care can also be provided in the context of a single consultation and through the whole care team being skilled in communication. In relation to the value of such care, both healthcare professionals and patients attributed many benefits to it including that it encouraged *'appropriate' *consulting behaviour, helped the patient feel more at ease in the consultation and to disclose more information, improved reported health outcomes, lowered care costs, and increased service provider and patient satisfaction. However, the authors note that the study involved a limited number of patients and health professionals. It also took place in one area of the UK, in Leicestershire, England.

Research evidence of the benefits of care with a personal component is usually defined as relational continuity (over time with the same healthcare professional). It examines benefits in terms of improved health outcomes and is contradictory in its conclusions. Some studies have found no association between continuity of care and improved health in relation to hypertension [15], gonorrhoea in teenagers [16] and epilepsy [17]. Other studies do indicate positive benefits in relation to the outcomes of care including, for example, in reducing patient medicalization and lower rates of hospitalisation [18,19], in reducing use of analgesia in labour and decreasing obstetric intervention [20], in saving time and using a more *'expectant management' *approach to care [21] and in greater uptake of preventive services [22]. There is also some evidence that relational continuity is associated with higher levels of patients' expressed satisfaction [23,24]. Those studies exploring the benefits of personal care in terms of the process and experience of care have found improvement in patient 'enablement' [3], as well as provider communication, patients' level of influence over care and their life satisfaction [25].

This paper presents data from a qualitative interview study on primary care in the UK, in Lothian, Scotland. It discusses patients' and GPs' perspectives on what receiving and providing personal care means to them and their experience of the benefits brought by it. Occasions when patients consulting with the known doctor were viewed as irrelevant or disadvantageous are also noted. The study focused on a different patient grouping from the Leicestershire study; participating patients were all parents with children aged up to ten years old, who gave accounts of their own healthcare and that provided to their families. The GPs were all from the practices of participating patients.

These findings are discussed in relation to current primary care research and organisational policy considering what should be included in definitions of personal care for the practical purposes of healthcare design and auditing of quality, especially with regard to the QOF, and the implications for future research.

## Methods

### Sampling

The study was undertaken prior to the introduction of the current contract in 2004. It used purposive sampling [26] to identify twenty-nine patients, mainly women with young children, and twenty-three GPs for qualitative interview using a semi-structured interview guide. Patients were all from participating practices but did not necessarily consult the doctors who took part. Linking participating patients with their own GP for the purpose of comparative analysis was considered unnecessary to the study aims and, by making the sampling strategy more complicated, would have made recruitment more difficult and time consuming. This sample group was selected in order to extend previous research evidence of adults' views on personal care, described above (13,14), by focusing on those of parents about their own healthcare and the healthcare provided to their young children (see Table 1 for details of participating patients) and by doing this in a different area of the UK. It also reflected the author's interest in families and children. Mothers are over-represented in the sample due to a lack of response to the study by fathers.

**Table 1:** Patient Characteristics

**Patient Reference **(All pseudonyms)	**Health Status**	**Practice Size**	**Mean Deprivation Score**
PP01-01 Mrs Thomson	No health problems	S	Low
PP01-02 Mr and Mrs Uphall	Parent with chronic conditions	S	Low
PP01-03 Ms Vickers	Parent with chronic stress-related problems/child with multiple disabilities	S	Low
PP01-04 Mrs Watson	Parent with chronic stress-related problems	S	Low
PP02-01 Mrs Peters	Parents with chronic conditions and 2 children with additional and complex needs	L	Low
PP02-02 Mrs Quinton	2 children with chronic conditions	L	Low
PP02-03 Mrs Richardson	Parent and 2 children with chronic conditions	L	Low
PP02-04 Mrs Smith	No particular health problems	L	Low
MP01-01 Mrs Anderson	Previous health problems with young child	S	High
MP01-02 Mrs Brown	Parent with chronic health problems	S	High
MP01-03 Ms Campbell	Parent with chronic health problem	S	High
MP01-04 Mrs Douglas	No particular health problems	S	High
MP02-01 Mr and Mrs Ewan	Both parents with chronic and debilitating health problems	L	High
MP02-02 Ms Forrest	Parent with chronic health problems	L	High
MP02-03 Mr George and Ms Grange	One parent with recent episode of ill-health	L	High
MP02-04 Mr Hendry and Ms Hamilton	Young child with chronic health problem	L	High
MP03-01 Mr and Mrs Inch	One parent with recent serious episode of ill-health	L	Low
MP 03-02 Ms Jackson	No reported health problems	L	Low
MP 03-03 Mrs Kyle	No reported health problems	L	Low
MP03-04 Ms Leishman	One parent with chronic health problems and child with recent health problems	L	Low
MP 04-01 Mrs Mooney	One parent had suffered from depressive illness	S	Low
MP 04-02 Ms Nichols	Recent stress-related illness and emergency care for children	S	Low
MP 04-03 Mr and Mrs O'Neill	Mother pregnant. No reported health problems	S	Low

S = small (3 GPs or less)

L = large (4 GPs or more)

PP = patients interviewed in the preliminary stage of the study

MP = patients interviewed in the main stage of the study

Since views on personal care may differ depending on practice size, GPs were sampled from large practices (defined as four or more doctors) and small practices (defined as three or less doctors). Relative deprivation may have influenced views so they were also sampled on the basis of Lothian Health Board's ranking of deprivation. Four practices were in three relatively deprived areas and three practices were in two relatively affluent areas (see Table 2 for details of participating practices). Patients with children aged up to ten years old were selected at random from each of the participating practices.

**Table 2:** Practice Characteristics

**Practice Reference**	**No GPs interviewed**	**Practice Size**	**Mean Deprivation Score**
P 1	3	S	Low -1.4
P2	2	L	Low -2.74
P3	1	L	High 4.25
M1	3	S	High 1.3
M2	3	L	High 1.53
M3	6	L	Low -0.7
M4	5	S (3 single-handed and 1 two-handed)	Low -1.8

S = small (3 GPs or less)

L = large (4 GPs or more)

P = GPs interviewed in the preliminary stage of the study

M = GPs interviewed in the main stage of the study

### Data Gathering and Analysis

Early interviews with nine patients (comprising eight interviews as one was a couple interview) suggested that the term 'personal care' used in the approach letter and explanatory leaflet had little salience to many patients. In addition, discussion of the concept of personal care, particularly when asked to give actual examples of times when they had and had not received what they considered to be such care, often resulted in patients discussing sensitive and private information. Upon reflection, it was decided to interview patients twice with a gap of around two weeks in between. This allowed participants to be more relaxed, reflect on what they had said in the first interview and to give greater detail overall in their accounts about the meaning and importance of personal care. Thus, the remaining twenty patients were interviewed twice (comprising thirty interviews as five were couple interviews). On a number of occasions patients had consulted their GP during the intervening two-week period and these very recent examples of care provided rich and detailed accounts of patients' experiences. Given the prominence of the concept of personal care in professional literature, and as these interviews were more difficult to arrange, twenty three GPs were interviewed once.

These sixty-one interviews in total were carried out using a topic guide that pursued the key research questions whilst also allowing flexibility for new areas of discussion to be raised by participants. Two slightly different topic guides were used for patients and doctors, both asking the same key research questions but with slight variations. In introducing the study to patients examples of the ways in which personal care has been conceptualised by others were given, whilst welcoming alternative possibilities. Both patients and GPs were also asked what they thought did not constitute personal care as negative instances also proved useful in elucidating how personal care was being defined. In addition, we were interested in whether and when receiving personal care held importance. Questions often resulted in patients giving detailed stories about times when it had been of value for themselves and their young children and in what ways, and when it had not been regarded as important. This also elicited information about patients' care of other family members. Both topic guides covered questions about practice organisation and whether there were any benefits or disadvantages to personal care. GPs were asked what facilitated and hindered them providing what they thought of as personal care.

Focusing with all participants on both what was and was not personal care and when it was and was not important, as well as asking for practical examples grounded in their daily experience, helped patients to orientate themselves to this otherwise abstract concept, to elucidate the meaning and importance of the personal in such care, and provided a safeguard against GPs merely replicating the well-entrenched rhetoric of the profession.

With interviewees' written permission, patients were interviewed at their homes and GPs at their practices. An information sheet of basic household details was completed for every patient at the end of the interview. All interviews were recorded and transcribed verbatim. The data were analyzed thematically, supported by the qualitative analysis software, NVivo, as well as more traditional paper-based methods. This entailed the study team reading and re-reading transcripts to identify recurrent themes, building a data categorisation scheme, and indexing data to this scheme whilst simultaneously interpreting their meaning in the context of the overall interview and other information about the participant. Framework Analysis [27], in which data are mapped on a matrix according to key themes as well as to individual characteristics, allowed a comparison between patients' and GPs' accounts and exploration of relationships between themes, practice size and relative deprivation. Constant comparison and systematic searching for deviant cases strengthened the process of analysis. Patients' and GPs' data were initially analysed separately and, upon comparison, were found to be significantly similar both at the overall thematic level and the level of more specific detail. A few differences in what patients and doctors emphasised in terms of the benefits and disadvantages of personal care are noted.

Data extracts used in this paper are typical of the comments made by participants and, so, are illustrative of the general themes that emerged from analysis.

## Results

### What Personal Care Means

Definitions of personal care were elicited through direct questioning and detailed accounts of examples of times both when they had and had not received or provided what was thought to be personal care (see both types of example in data extracts below). It was talked about in three different ways by both patients and GPs: As resulting from the practitioner having a particular consultation style (*personal consultation style*); through the whole practice providing accessible, friendly and well co-ordinated care (*whole practice *care); and as the result of care which occurs within a relationship with one or a small number of practitioners built over time, one crucial characteristic of which is that the patient knows and is known by the doctor (*relational continuity*).

Whilst personal care was described in these three ways, providing or receiving care experienced as personal was rarely conceptualised exclusively one way or the other. Usually participants talked about them, not as distinctive but, as related concepts. Care given by the known doctor with whom an interpersonal connection had been established was overwhelmingly the main way that participants discussed how personal care was provided. However, consulting the known GP by itself was not thought necessarily to result in care they deemed personal. Seeing the same practitioner who did not have a particular consultation style and with whom there was no interpersonal connection usually was not regarded as personal care. Only a few participants regarded personal care as being possible within the context of a single encounter even when such a consultation style was used. *Whole practice care *was considered by some participants to be a way through which personal care could be provided. However, on the whole, this was not regarded as essential to or a substitute for the main way that personal care is provided; by the known practitioner who has a particular consultation in the context of a trusting relationship in which patient and doctor know one another.

Patients from large (and, of course, single-handed) practices were more likely to refer to having an exclusive relationship with one doctor, whilst those from small practices tended to refer to having a relationship with more than one practitioner. This difference may be due to the fact that in smaller practices patients have a greater chance of seeing all the doctors for the logistical reason that surgeries must be shared by two or three practice partners. It seems reasonable to suggest this enables patients to get to know and be known by all of the doctors in the smaller setting and to build a trusting relationship with them in a way not so possible within the larger. However, within smaller practices, the emphasis on an exclusive relationship as an essential facet of personal care was notably greater when the patient or a member of her family suffered a complex, serious and worrying problem. In these situations of high emotional salience, patients in small practices usually expressed personal care as occurring through the context of a relationship over time with one specific doctor. No differences were found in how patients defined the meaning of personal care with respect to relative practice deprivation.

Specific features were regularly mentioned as being important in providing care that was experienced as personal. Both patients and GPs talked about all practice staff having a particularly pleasant and approachable manner and attitude towards the patient, but especially that of the consulting practitioner. They also mentioned the patient being dealt with as an individual whose treatment is tailored to her particular circumstances and needs. Most patients and all GPs thought treating the patient as an individual required the doctor to hold knowledge about the patient as an individual (previous medical history, response to medications, personality and preferences), as well as wider family and social/economic circumstances. Both participant groups thought that having an interpersonal connection with the practitioner who knows the patient and who has a commitment to her general wellbeing were also important to care being personal. Generally, it was couched by patients in terms of the patient seeing *my own doctor, the usual doctor, my family doctor*, and it frequently included a sense of the patient identifying and sharing an interpersonal connection with the doctor who knows them. As noted, seeing the same doctor over time without this connection and the feeling of knowing and being known was not considered to result in care being personal care.

The following extracts from patients' data illustrate some of these points regarding the meaning of personal care:

'Personal care... would be one doctor, who personally looks after me, and he would be the only one person that I would see. That's what I would take it to be. So, they would really know me' (MP01-04).

'... I think the main thing is that [the known GP] has been there for me in the past... it's nice to know... that your GP's there... that he's really interested in what's happening with you... and caring about your health... and concerned about your well-being, you know, it's nice to know that somebody's there... [Personal care is not when] ...my son took ill with epilepsy... he had that first one... through the day and went on to have another two that evening, and I was really distraught, you know, really frantic, and he [the unknown doctor] came out and said, "Well, it's only a fit", and I was saying, "Well, you know, there's something wrong, this isn't the normal pattern for Douglas, there's got to be something wrong here...". But, in the end, it was a case that my son is going into hospital because there is something wrong, but he was like, "Well, it's just a fit"...' (PP 02-01).

'It [personal care] is not something that would necessarily follow [from always seeing the same doctor] ... if the doctor doesn't make someone, it has got to come from the doctor, too, of relating to the person they're dealing with... if you've got the kind of doctor who is very aloof and not really listening, he could see you twenty times and it wouldn't make any difference because they're not interactive...' (PP01-01).

These extracts from doctors' data similarly illustrate these points regarding the meaning of personal care:

'Well, [personal care is] I think the essence to me of what general practice is, is that you have a relationship with a patient over time and, if you've been their GP for a number of years' (PD01-02).

'I suppose by definition..., I was thinking about personal care, and I was thinking very much in terms of continuity of care and length of relationship and things' (MD03-03).

'...it's an ongoing commitment really, it's not a one-off consultation with an individual patient, but it's kind of something over a prolonged period of time or for which the doctor feels responsible for that patient, outwith a particular consultation. It also means being available, the patient knowing that the person they go to who will take that responsibility for it... It's a kind of ongoing commitment really' (PD01-01).

' [Personal care is not] ignoring their agenda, dealing with only the technical side, not the personal, the emotional side, making presuppositions about them, I mean, labelling them, for instance, failing to negotiate. I think, like I said before, failing to develop any mutual respect or empathy, whatever that might be. It doesn't have to be affection. I mean, ideally, there should be an element of affection, but some sort of respect of the situation they're in and how they're dealing with it' (PD 03-01).

### The Benefits of Personal Care

Numerous benefits were ascribed to personal care usually when defined as seeing the known doctor. This is implicit in the way participants talked about what providing and receiving such care means to them (see above data extracts). Most regarded it as an important aspect of high quality care. Patients and GPs reported experience of similar benefits relating to quality of care in terms of, first, improved *health outcomes *and, second, the *experience *of seeking, receiving and providing health care.

### Health Outcomes

Patients and GPs reported health outcome benefits including that the doctor's greater knowledge of the context of people's lives led to quicker, more accurate diagnosis and more individualized and efficacious treatment. It was also thought to improve the ability of both patient and doctor to monitor and manage a treatment plan. Both sets of participants reported more patient participation in the consultation, as well as greater patient trust in the doctor, and that this led to the patient being more involved in, and committed to, the treatment plan. In addition, doctors reported that personal care supported them to arrange fewer medical investigations and hospital admissions and to prescribe less, whilst taking fewer risks than with unknown patients. This was regularly compared favourably by GPs to work within the out-of-hours service.

### Experience of Seeking, Receiving and Providing Care

The benefits of personal care reported by patients and GPs relating to the experience of seeking and receiving care included that patients could more easily consult about some (though not all) personal concerns. They stated that both time and effort were saved due to the doctor having assumed, shared knowledge of the patient that does not have to be repeated. They agreed that it led to patients generally feeling more comfortable during, and being enabled to participate more in, consultations. Patients were thought to be taken more seriously and to be believed by the known doctor.

Generally, personal care was considered to attend well to the emotional or affective aspects of the experience of seeking and receiving healthcare. In particular, it was described mainly, but not exclusively, by patients as allowing the patient to be known as a competent person with a legitimate claim to service, as someone who would not 'waste' the doctor's time. Patients further emphasised how it enabled doctors to know them as competent parents with expert knowledge of their children. This was clearly of importance to patients as it reassured them during stressful times when they were concerned about their children's health or that of other adults that appropriate medical care would be provided. This was described as resulting in them having their healthcare expectations for themselves and their families better met and generally in being provided with a higher quality service for the whole family. Some patients reported that it sometimes led to ease of access for more urgent care.

In addition, doctors contended that personal care helped them to encourage the patient to *'open up'*, for them to *'get behind the presenting problem'*, and to better negotiate an agreed plan of action with the patient which the patient would then be more likely to carry out. GPs noted that providing personal care is what gives them a sense of vocational satisfaction (although it is also reported to place a strain on their own internal resources). Further, they reported that it enabled them to better manage those patients deemed 'problematic'.

In respect of both areas of benefits ascribed to personal care, no differences were noted according to either practice size or deprivation score.

The following extracts from patients' data illustrate some of these points regarding the benefits of personal care:

'Eh, the doctor knows the two of them [her daughters] inside out... she knows it's going to be either 'A' or 'B' between asthma and periods... you're only in about five minutes because you don't have to go through the whole history of them... I think that is a major advantage because when I work full-time, I don't have time to sit for half-an-hour in a doctor's surgery. If you just go in, get seen, get what you want and out the door... Yes, I think it is just the family unit and you want the family unit to be stable... he's got problems [her husband], the two kids have got problems... I just like the family unit to be stable and whoever goes down to the doctor is going to come out with the right answer' (PP 03-01).

'That's if we get the same doctors ... if you get a different doctor, you go, "He's (young son with asthma) wheezy", that's it... or you are so uptight because you are so panicky. Where, if you get your normal doctor, you can relax and go, "Right, this is what has happened..."' (MP02-04).

'I feel comfortable when I do go to those two doctors [her known doctors]. But, if I go to another doctor, eh, you know, I feel uncomfortable. Like, quite a few times I've had a different doctor, eh, I felt uncomfortable, "What can I say to them, like?" But, with they doctors, I just sit there, go in, just sit comfortable and get really comfy. I feel comfortable with they doctors, but with the other doctors I just dinnae feel comfortable' (MP 01-03).

'... somebody who knows you and believes you,..., if you go to a GP that you don't know, they don't know if you are making it up, or exaggerating, and I have a sister who has never got anything wrong with her, and she's a chronic, "Oh, I've got a terrible headache, oh, I feel awful". You feel like saying to her, "There's really nothing wrong with you". It's not that there's nothing wrong with her, but she moans a lot about it, so I suppose the GP knows who's like that or who is quietly saying, "I've got a bit of a headache", and has actually got meningitis...' (PP01-01).

The following extracts from doctors' data similarly illustrate some of these points regarding the benefits of personal care:

'Well, the example again would be the mums. ..."Wee Jamie's not well", you know, "He has a temperature", or whatever. If I knew it was [name of patient], I know she's a caring mum who's got two kids, lives down the street and she's got a nanny and, you know, she hasn't phoned up for a year... I would take this pretty seriously because she never phones up, she's a sensible mum, she wouldn't phone unless she was worried about it. Now, after-hours you get half a dozen plus of these calls. You can make judgments... on people, the way they speak, their address, we have to do that...' (MD04-05).

'I think... if you have to... break bad news, for example, or if you perhaps decide to take a line of management the patient wasn't expecting, I think it's probably much easier to reach agreement with the patient and his family if you know them well than if you don't know them at all' (MD03-04).

'... [not giving personal care is] defensive medicine, you have to cover everything... it's dangerous and uncomfortable, really... where patients go to see somebody new and, then, quite often they get shunted off into all sorts of investigations or tests or treatments or things which I think, in retrospect... were inappropriate really... because of fear of missing something' (PD01-01).

'... if they came in off the street, you would tend to be more concerned about symptoms ...do more tests. ... [In out-of-hours] I'm sure there's a lot more intervention done. ...we get sheets back and you think, "Oh, we didn't need to do that" because, if somebody's seen them cold they would be more concerned about things that we would say, "No, that's alright". [If] you don't know the people at all... not knowing the history... you're pressurized [and this] probably leads you to admit more people... their own GP wouldn't necessarily admit them' (MD03-06).

### When Personal Care is Valued

Having the choice of consulting with the known and trusted GP who adopted a particular consultation style generally was deemed to be very important by both patients and doctors. However, participants described circumstances when it was considered very important and others where it was considered less so. Seeing a known doctor was sometimes traded-off against competing priorities or was deemed irrelevant and, in a few cases, it was thought to be undesirable.

As indicated by the above extracts, participants described personal care as being at a premium when patients are suffering from ambiguous or complex problems. It was also thought to be important when a diagnosis is elusive or if patients have multi-faceted, psycho-social or long-term mental health problems. Patients suffering from physical conditions that are chronic, serious, life-threatening or terminal, and for some, though not all, problems of a more personal nature were reported to place a high value on experiencing personal care. Pregnancy was reported to be a time when personal care had a high level of importance. Patients also stated it had particular importance when in a caring role, either as parents of young children or with adults suffering from chronic and debilitating health problems. For instance, such care was thought to be of particular importance when parenting young children as the known GP would be more likely to believe and take them seriously, resulting in their children receiving the care they needed. It was, in effect, described as being one element that supported and sustained family life, especially in difficult life circumstances.

Most patients and doctors reported personal care to be less important for acute and also for more common, everyday problems. Patients stated that they regularly traded-off such care for speed of access to any healthcare practitioner for emergencies or for minor and self-limiting illnesses where *'any doctor will do'*. Doctors agreed that personal care has lesser importance for patients in these situations. However, many thought that seeing patients in these situations was still useful for building up a relationship with patients and, so, could contribute to their ability to provide care they experience as personal. Patients prioritising speed of access over seeing the preferred doctor was particularly evident amongst those registered with large practices where more trading-off activity was described than in small ones. This may be explained by the logistical problem of surgeries being held by a larger number of (potentially unknown) doctors than is the case in smaller ones. No differences were noted in this respect by practice deprivation score.

Patients described some situations where seeing a known doctor was undesirable, including for some embarrassing or intimate problems such as gynaecological examinations where gender of doctor was an issue or about sexually transmitted diseases. The problem was identified as being known by and knowing the doctor too well. Patients sometimes wanted to maintain certain boundaries around how they were known by the GP(s) from whom they usually sought personal care. At such times, patients seeing a doctor in the practice with no previous contact with them or having anonymity from another part of the healthcare system, such as a Well Woman Clinic, was preferred. Doctors also acknowledged that providing personal care to patients, whilst being the source of their vocational satisfaction, also carries the risk of them feeling stressed and 'burnt-out', particularly when dealing with patients considered 'problematic'.

The following extracts from patients' data illustrate some of these points regarding when personal care was and was not valued:

'...if it was to do with a lump in my breast, or something like that, I think I would prefer to go and see either [own doctors] because that's the two I'm really close with. ... if I found a lump... or if I felt I was having a miscarriage or something like that, yes, I would definitely go to [doctor who knows her] about those things, more in-depth. But, if it was just a case like... back pain or something, then, I would go to any doctor.... If it was something more personal to do with myself or to do with depression..., then I would prefer to see my own doctor' (MP02-01).

' [Personal care is important] when you have children, in particular... because one of the things that I think that's based on is the fact that the doctor has to trust me. I'm a parent and I know my children inside out, and I know if there's something wrong... so, I think that it's very important that the doctor has to trust my judgements... So...I feel if they trust me to know when... somebody should be seen by the doctor, then that also has to be reciprocated, if they say to me, "Oh, she's fine, just do this, do that, she's ok, don't worry", then I believe them, because the trust is a two way thing' (MP 04-02).

'...where you think it's just a viral infection and temperature and being sick, it would be more important to see any doctor that day because I wouldn't wait for a particular doctor and things like, yes, can you confirm this is chickenpox? ... I don't think it would matter which doctor' (MP03-04).

'....It [seeing a doctor who doesn't know her] could be useful for something where you wanted to be anonymous, if you had a sexually transmitted disease or something.... And, equally, for me, if there was anything like that that I didn't want to go to my GP for, I would go to the Well Woman Clinic..., it's [seeing the known doctor] more just uncomfortable' (MP04-02).

The following extracts from doctors' data similarly illustrate some of these points regarding when personal care was and was not valued:

'For people with chronic problems it's [personal care] really important... I suppose the elderly, just partly because they tend to have more things going on, em, people who have lots of appointments about the same thing, you know, for ante-natal patients, for instance' (MD 03-06).

'I suppose there's probably a group of patients for whom not having personal care is completely ok, ...people, who come in for episodic things... "You've got a sore throat. Yes, it will take time to get better. No, there isn't much I can do about it", the sort of easy reaction to the symptom, prescription and kind of out of the consultation...' (MD 04-01).

'Mainly, [personal care is most important] if someone has been managed for illnesses that might have a psychological component, mental depression or any of the chronic conditions... usually things that require the art of medicine as well as the science. You know, someone comes along and they've got tonsillitis, and... it's just like having a car serviced, if you like, it doesn't matter who changes the clutch. But, if you have to make a diagnosis or management which involves more complex, psycho-social element things, I think it should be the same person...' (MD02-02).

'...one patient I saw every few weeks for years who had a big drug problem and who could be incredibly aggressive ... and I'd go home in the evening, I would feel completely drained, like he'd sucked out all the life-force out of you.... I don't know how to express that, really, because it's a personal resource that's drawn out of you in the process..., which is very satisfying as well... but, you also feel kind of burnt-out, slightly' (PD 01-01).

## Discussion

This study suggests that personal care was mainly defined, experienced and valued as care provided in the context of an ongoing relationship in which a particular consultation style is shown and which is characterised by the patient and doctor knowing one another and sharing a mutual connection. A few participants also described personal care as being possible from the whole care team, as well as within a single consultation, suggesting that these facets should be included in any working definitions and models of care developed. In this respect, this study concurs with that undertaken in Leicestershire (13, 14) and, as it was carried out in a different area of the country with a different patient grouping, lends weight to these findings.

However, this study further suggests that these contexts from which personal care can be given are not mutually exclusive. Seeing the known practitioner over time who does not have a particular consultation style and with whom no interpersonal connection has been established is not considered to result in personal care. Likewise, it shows that care experienced as personal provided by the whole team and within a single consultation, though important, should not be regarded as an alternative to the potential for patients to build relationships with one or a small number of doctors. The continuing importance placed by participants on personal care defined in this important way is clear and concurs with findings from other studies [19,28]. Consequently, policies and organisational arrangements that support and give incentives to the provision of care in the context of a relationship should remain or, where necessary, be put in place.

The study also contributes to our knowledge about the potential benefits of personal care defined in this way. Significant benefits were reported by patients and practitioners as relating to both improved health outcomes and patients' overall experience of care. These data are based on self reports but the views expressed concur with the findings of other studies that have used a mixture of research methods [14,18-20,22,25]. The evidence suggests that personal care, usually defined as care provided in the context of an ongoing relationship, *may *result in improved diagnosis, more efficacious treatment, reduced medicalisation, reduction of risk, greater patient enablement in the consultation and patient agreement with and adherence to the treatment plan.

An additional finding of this study is that participants emphasised its value in relation to improving the affective elements of patients' experience of seeking and receiving health care. Seeking and receiving care can be emotionally challenging, especially when a patient or someone in the family is facing health issues that are complex, debilitating and sensitive in nature and which provoke considerable uncertainty and anxiety. Personal care, and knowing and being known by the doctor as an integral aspect of this, was experienced as attending to such heightened emotions in a way that patients greatly valued as helping to smooth their path through the healthcare system.

Seeking and receiving care also place patients in a morally ambiguous position. Patients' concern to emphasise not only the importance of being known, but of being known *in a particular way *as a trustworthy person with a legitimate and deserving claim to service, indicates their awareness of the moralising context within which they must operate when attending to their health concerns. Personal care was said to facilitate patients to negotiate their way through this morally-suffused health care system, to obtain the care they deemed necessary and, more generally, to manage the experience of illness in the family. Through consulting the known practitioner, patients did not have to re-negotiate their moral identity and this was described as reducing the risk and anxiety they experienced when consulting. In particular, parents of young children for whom being believed and taken seriously, being known as a competent parent who knows best when their child needs medical care, is of crucial importance.

This may explain, in part, why personal care was reported to have particular salience to patients when facing conditions that provoke heightened emotion, for instance, suffering from chronic and debilitating ill-health and during certain life stages, such as when pregnant, parenting young children or in other familial caring roles and when facing generally difficult life circumstances, including being socio-economically disadvantaged.

Participants valued personal care because it improved their health outcomes. They also perceived it enabled them to manage their identity and so better negotiate about their health care. As they equated these with high standards of care, both aspects should be reflected in measures of quality when designing and auditing primary care services.

However, noting the disadvantages of providing and receiving personal care reported by both patients and doctors is also of importance. Some patients did not want to consult with the known practitioner for problems considered embarrassing or intimate suggesting that they did this in order to preserve their identity as legitimate and deserving patients. Likewise, doctors talked about the stress caused by providing personal care to patients they deemed problematic. This raises a question about how much personal care, as well as the quality of such care, is being provided to those patients who are perceived in a negative way.

The study is limited in that it was undertaken in one area of Scotland. GPs who took part became involved out of their commitment to this approach and may not be representative of the profession overall. As the patient sample was drawn from parents with at least one child up to ten years old, most were aged twenty to fifty years, and women were over-represented.

Nevertheless, these findings generally support the Leicestershire study that personal care has continuing salience for both patients and GPs in the modern UK primary care. The study suggests that the move toward a public health agenda, what might be called the 'public health patient' and 'technocratic doctor', and that may have been consolidated by the GP contract, is out of step with these findings. The contract awards the majority of 'points', and associated financial remuneration, to activities related to clinical outcomes and practice organisation (up to 950 of a possible 1050) and demonstrates the emphasis being placed on purely clinical, technically-oriented care within primary care. The contract awards only up to 100 points for aspects of care usually associated with personal care (continuity, relationship, interpersonal skills and length of consultation) illustrating the relatively lower value being attached to these aspects of service. Further, the only part of the contract dealing with personal care is in the form of patient surveys. Points awarded are not related to the results of these surveys and there is currently no obligation or additional financial incentives for practices to improve services based on results.

It is concerning that, whilst the evidence suggests personal care has greatest importance for those suffering from chronic and debilitating health problems and their families, the contract incentivizes specialisation and fragmentation of services for this group rather than the *relational continuity *combined with the particular *consultation style *so valued by those in our study and related research.

## Conclusion

The evidence of this study is that participants valued care in which patients are dealt with as individuals whose wider domestic and social circumstances may be taken account of, so that treatment can be tailored to meet their specific needs within their life context. It also contributes to evidence that patients may benefit from personal care in terms of improved medical outcomes and in being facilitated through the process of care including through being known in a particular way.

No doubt, internal reviews and empirical research assessing the impact of the contract are being planned and undertaken currently. Given the above, amongst the issues needing to be addressed are: whether population level medicine is superseding personal care and is leading to a diminution in some aspects of quality; assessment of the extent to which practices have used patient surveys and the changes effected as a result; whether those practices trying to maintain personal care are being discriminated against financially, devalued and demoralised within this framework; and what the actual consequences are for the most vulnerable groups, for example, those with chronic, multiple and debilitating conditions and those in lower socio-economic groups, as well as those who are deemed 'problematic' patients. It may be necessary to redefine and give incentives to improve access to care, not as speed of access to the first available practitioner but to the most 'appropriate', including the known, practitioner. Audit and research both should be designed to answer these questions as the contract and QOF come under operational scrutiny and review.

## Competing interests

The author declares that she has no competing interests.

## Authors' contributions

RA undertook this study for her PhD at the Department of General Practice, The University of Edinburgh. She was the principal researcher in all stages of the study and wrote this paper.

## References

[B1] Berger J (1969). A fortunate man, the story of a country doctor.

[B2] Loudon I (1984). The concept of the family doctor. Bulletin of the History of Medicine.

[B3] Howie JGR, Heaney DJ, Maxwell M, Walker JJ, Freeman GK, Rai H (1999). Quality at general practice consultations: cross-sectional survey. British Medical Journal.

[B4] (2001). Scottish Executive. NHS Plan for Scotland.

[B5] British Medical Association General Practitioners Committee (2000). Shaping Tomorrow: issues facing general practice in the new millennium. London.

[B6] Starfield B (1980). Continuous confusion?. American Journal Public Health.

[B7] Freeman G, Hjortdahl P (1997). What future for continuity of care in general practice?. British Medical Journal.

[B8] Tuckett O, Boulton W (1985). Meetings between Experts.

[B9] McArthur CA (2002). Counting beans.. British Medical Journal.

[B10] Taylor MB (1997). Compassion: its neglect and importance. British Journal of General Practice.

[B11] Martin R (2004). Linking physicians' pay to quality of care – A major experiment in the United Kingdom. New England Journal of Medicine.

[B12] Heath I (2004). Access to primary care and social inequalities. The Future of Access to General Practice-based Primary Care – Informing the Debate.

[B13] Preston C, Windridge K, Baker R, Freeman G, Boulton M (2001). Should primary care groups act to improve personal care?. Report from the Clinical Governance Research and Development Unit.

[B14] Tarrant C, Windridge K, Boulton M, Baker R, Freeman G (2003). Qualitative Study of the meaning of personal care in general practice. British Medical Journal.

[B15] Phillips D, Shear C (1984). Provider continuity and control of hypertension. Journal of Family Practice.

[B16] Chacko M, Wells R, Phillips S (1987). Test of cure for gonorrhoea in teenagers. Who complies and does continuity of care help?. Journal of Adolescent Health Care.

[B17] Freeman G, Richards S (1998). Personal continuity and the care of patients with epilepsy in general practice. British Journal of General Practice.

[B18] Gill J, Mainous A (1998). The role of provider continuity in preventing hospitalizations. Archive of Family Medicine.

[B19] Menec V, Sirski M, Attawar D, Katz A (2006). Does continuity of care with a family physician reduce hospitalizations among older adults?. Journal of Health Services Research & Policy.

[B20] Flint C, Poulengeris P, Grant A (1989). The 'know your midwife' scheme: a randomized trial of continuity of care by a team of midwives. Midwifery.

[B21] Hjortdahl P, Borchgrevink C (1991). Continuity of care: influence of general practitioners' knowledge of their patients on use of resources in consultations. British Medical Journal.

[B22] Doescher M, Saver B, Fiscella K, Franks P (2004). Does continuity count?. Journal of General Internal Medicine.

[B23] Baker R, Streatfield J (1995). What type of general practice do patients prefer? Exploration of practice characteristics influencing patient satisfaction. British Journal of General Practice.

[B24] Baker R (1996). Characteristics of practices, general practitioners and patients related to levels of patients' satisfaction with consultations. British Journal of General Practice.

[B25] Love M, Mainous J, Talbert Hager G (2000). Continuity of care and the physician-patient relationship; the importance of continuity for adult patients with asthma. The Journal of Family Practice.

[B26] Mason J (1998). Qualitative researching.

[B27] Ritchie L, Spencer L, Bryman A, Burgess RG (1994). Analyzing qualitative data.

[B28] Grol R, de Maeseneer J, Whitfield M, Mokkink H (1990). Disease-centred versus patient-centred attitudes: comparison of general practitioners in Belgium, Britain and the Netherlands. Family Practice.

